# Radiation-Tolerant *Fibrivirga* spp. from Rhizosphere Soil: Genome Insights and Potential in Agriculture

**DOI:** 10.3390/genes15081048

**Published:** 2024-08-09

**Authors:** Sathiyaraj Srinivasan

**Affiliations:** Department of Bio & Environmental Technology, College of Natural Science, Seoul Women’s University, Seoul 01797, Republic of Korea; drsrini@swu.ac.kr; Tel.: +82-2-970-5670

**Keywords:** biofertilizer, *Arabidopsis*, genome, siderophore, PGPRs

## Abstract

The rhizosphere of plants contains a wide range of microorganisms that can be cultivated and used for the benefit of agricultural practices. From garden soil near the rhizosphere region, Strain ES10-3-2-2 was isolated, and the cells were Gram-negative, aerobic, non-spore-forming rods that were 0.3–0.8 µm in diameter and 1.5–2.5 µm in length. The neighbor-joining method on 16S rDNA similarity revealed that the strain exhibited the highest sequence similarities with “*Fibrivirga algicola* JA-25” (99.2%) and *Fibrella forsythia* HMF5405^T^ (97.3%). To further explore its biotechnological potentialities, we sequenced the complete genome of this strain employing the PacBio RSII sequencing platform. The genome of Strain ES10-3-2-2 comprises a 6,408,035 bp circular chromosome with a 52.8% GC content, including 5038 protein-coding genes and 52 RNA genes. The sequencing also identified three plasmids measuring 212,574 bp, 175,683 bp, and 81,564 bp. Intriguingly, annotations derived from the NCBI-PGAP, eggnog, and KEGG databases indicated the presence of genes affiliated with radiation-resistance pathway genes and plant-growth promotor key/biofertilization-related genes regarding Fe acquisition, K and P assimilation, CO_2_ fixation, and Fe solubilization, with essential roles in agroecosystems, as well as genes related to siderophore regulation. Additionally, T1SS, T6SS, and T9SS secretion systems are present in this species, like plant-associated bacteria. The inoculation of Strain ES10-3-2-2 to *Arabidopsis* significantly increases the fresh shoot and root biomass, thereby maintaining the plant quality compared to uninoculated controls. This work represents a link between radiation tolerance and the plant-growth mechanism of Strain ES10-3-2-2 based on in vitro experiments and bioinformatic approaches. Overall, the radiation-tolerant bacteria might enable the development of microbiological preparations that are extremely effective at increasing plant biomass and soil fertility, both of which are crucial for sustainable agriculture.

## 1. Introduction

The rhizosphere is a key zone where plant roots interact closely with various microbial communities. This interaction zone is a dynamic and complex ecosystem heavily impacted by various chemical molecules that are found in root exudates, including sugars, amino acids, and secondary metabolites [1]. In addition to providing energy for microbial metabolism, these exudates facilitate complex interactions between bacteria and plants, affecting the composition and activities of microbial communities. The rhizosphere is essential for nutrient uptake, disease resistance, and stress tolerance in the context of plant physiology [2]. This zone’s microbial residents can break organic matter, increase nutrient availability, and either suppress or compete with pathogens. Additionally, they directly impact plant growth and the sustainability of ecosystems by playing critical roles in the biogeochemical cycles of nitrogen, phosphorus, and other important elements [3].

The significance of the rhizosphere in agroecosystems has been underscored by the latest developments in molecular biology and metagenomics, which have started to reveal its extensive and intricate network of interactions. Rhizobacteria, for instance, could control plant hormone levels, affecting how plants develop, including how their roots are arranged and how their shoots grow [4]. Plant-growth trajectories are impacted by this microbial regulation of plant hormones, which also improves plant resistance to abiotic challenges such as salt, drought, and heavy metal toxicity [5]. Agricultural innovations should focus on the rhizosphere because of its vital role in maintaining plant health and productivity. By utilizing rhizosphere dynamics, farming operations can become more sustainable, lowering the need for chemical inputs and increasing crop yields in various environmental settings.

Due to its enormous energy and strong penetration, γ radiation is a major environmental stressor that substantially affects biological systems. γ rays, which are produced mainly by nuclear decay, cosmic sources, and human-made processes like nuclear reactors and medical radiography, can seriously harm biological macromolecules, especially DNA and proteins [6,7]. When γ rays interact with biological tissues, free radicals can cause molecular damage, oxidative stress, and cell death. Despite these challenges, a particular group of bacteria, referred to as extremophiles, display extraordinary adaptations that enable them to endure high radiation levels and flourish. Microorganisms, such as bacteria, fungus fungi, and archaea, have evolved a range of biochemical and molecular defenses against the deleterious effects of radiation. For example, certain bacteria possess extremely potent DNA repair systems that allow them to repair radiation-damaged DNA quickly. Instead, a few maintain high levels of antioxidant enzymes to lessen oxidative stress [8]. Furthermore, these microbes’ adaptable mechanisms are particularly noteworthy considering the changing global climate and the rise in the frequency of extreme weather events [8]. Creating strategies based on knowledge of how organisms adjust to radiation and promote plant growth can increase the resilience of agricultural systems against environmental stressors.

The Type I, VI, and IX secretion systems (T1SS, T6SS, and T9SS) are potent bacterial molecular weapons that can inject effector proteins into prokaryotic or eukaryotic cells, allowing bacteria to compete and adapt to their surroundings more efficiently. Although most of the current T6SS research has focused on bacteria, this system is also essential for plant-associated bacterial adaptability [9,10]. Considering these agricultural challenges and opportunities, this study focuses on the isolation and characterization of a bacterium from rhizosphere soil subjected to high levels of γ radiation. This isolation process targets the microbial survivors of 3kGy γ irradiation [7,11], a dose that significantly challenges microbial viability and selects exceptional resilience traits.

Organic farming, which uses biofertilizers, has become popular globally in sustainable agriculture as a response to the growing demand for safe and healthful food and long-term sustainability. It increases soil biodiversity in addition to guaranteeing food safety [12]. The ability of *Fibrivirga* sp. to tolerate radiation could be particularly useful in environments where radiation levels might be higher than usual, such as in contaminated sites. The key benefit of biofertilizer is that it has a longer shelf life and does not negatively affect the ecology. Applying compost, biofertilizers, biopesticides, and other products is part of organic farming. At the same time, they might not be a perfect replacement, but they can be a valuable addition to reducing the use of agrochemicals. The primary microbiological flora of soil, which includes all forms of eubacteria, archaebacteria, eukarya, and fungi, including the plant-growth-promoting rhizobacteria (PGPR), makes organic farming possible [13]. Many PGPRs comprise some of the main components for plant growth: siderophores and auxin. According to Neilands [14], siderophores, or “iron carriers”, are low-molecular-weight (500–100 dt), ferric-ion-specific chelating agents produced under iron-limited conditions by a variety of PGPR.

Within the phylum Bacteroidetes, the genus *Fibrivirga* is a member of the order Cytophagales and the family *Spirosomaceae* [15]. According to the List of Prokaryotic Names withstanding in Nomenclature (https://lpsn.dsmz.de/family/spirosomaceae; accessed on 4 April 2024) and Bergey’s Manual, this family now consists of 26 genera, which has the genus *Fibrivirga* among them. This study is an initiative to focus on radiation resistance and plant growth within this group of bacteria. This study encompasses identification, genomic sequencing, and functional assays to explore the genomic underpinnings of radiation tolerance and plant-growth promotion. The genome sequencing aims to identify specific gene clusters responsible for these traits, providing insights into the molecular mechanisms that enable this bacterium to thrive under the dual stresses of radiation and agricultural production pressures. Through understanding the adaptive mechanisms of this γ-resistant *Fibrivirga*-related strain, we aim to expand the toolkit available for developing resilient agricultural systems capable of withstanding multifaceted environmental stresses.

## 2. Results

### 2.1. Strain Isolation and Identification

**Figure 1:**
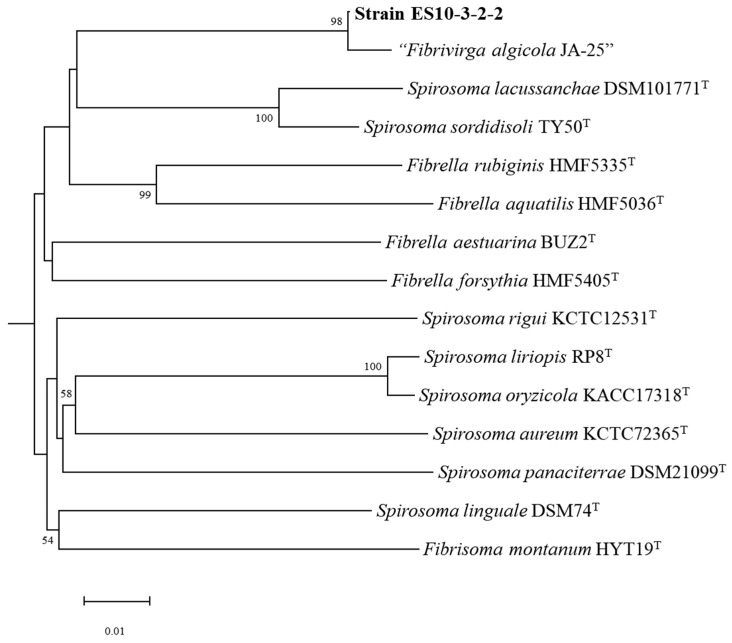
The genome-based phylogenetic tree of ES10-3-2-2 and its related type strains determined using data from the Type Strain Genome Server. The phylogenetic tree was constructed using the calculated intergenomic distances to infer a balanced minimum evolution tree. This analysis utilized the FASTME v.2.1.6.1 software, incorporating Subtree Pruning and Regrafting (SPR) post-processing [17] to refine the tree topology. Branch support was determined through 100 pseudo-bootstrap replicates. The resulting trees were midpoint-rooted [18] and visualized using MEGA (v.8.2) software.

Strain ES10-3-2-2 was isolated from an irradiated garden soil sample collected at Seoul, and the cells were Gram-negative, aerobic, and non-spore-forming rods that were 0.3–0.8 µm in diameter and 1.5–2.5 µm in length (Supplementary Materials, Figure S1). The colonies were circular, translucent with an entire margin, and pale-pink-colored on an R2A agar medium. Three copies of the 16S rRNA gene found in the genome were identical (1510) and compared with the closely related taxa obtained from GenBank. Strain ES10-3-2-2 was considered to belong to the genus “*Fibrivirga*” (not validly published according to LPSN (https://lpsn.dsmz.de/genus/fibrivirga; accessed on 4 April 2024)) with the closest relatives being “*F. algicola* JA-25” [16] (99.2%) and *F. forsythia* HMF5405^T^ (97.3%). The genome-based phylogenetic tree (Figure 1) showed the Strain ES10-3-2-2 monophyletic clade with “*F. algicola* JA-25”. The pure culture of Strain ES10-3-2-2 was deposited at the NITE Biological Resource Center (NBRC) under the accession number NBRC 115440. Based on the 16S rRNA (Supplementary Materials, Figure S2) and genome-based phylogenetic analysis, the newly isolated strains belong to the genus *Fibrivirga* of the family *Spirosomataceae* in the phylum *Bacteroidota*.

### 2.2. Genome Sequencing and Analysis

#### 2.2.1. Genome Properties

The genome of Strain ES10-3-2-2 consists of a circular chromosome of 6,408,035 bp with a GC content of 52.8%. A total of 5252 genes were predicted, including 5038 protein-coding genes, 52 RNA genes, and 45 pseudogenes. A total of 2944 genes were assigned to have putative functions, and the remaining genes were annotated as hypothetical or converted hypothetical proteins. The sequence assembly also revealed three complete plasmids with the size of 212,574 bp, 175,683 bp, and 81,564 bp. Sequence project information showing the genomic details is shown in Table 1.

**Figure 2:**
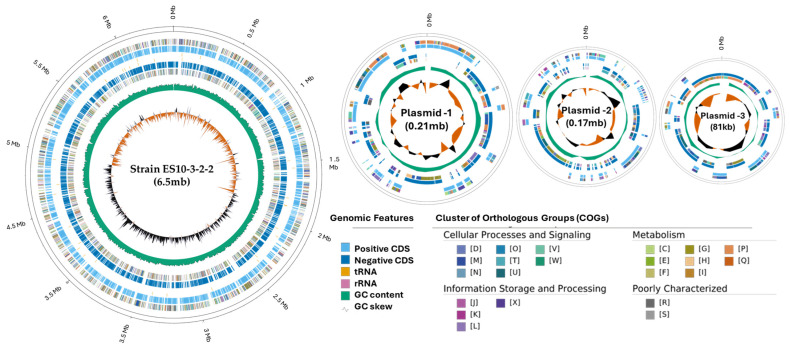
Circular map of the Strain ES10-3-2-2’s chromosome and plasmids. The outer circle shows the scale in metabases (Mb). The representations, from the outer to the inner circle, are forward- and reverse-strand CDSs.

A total of 5809 genes were categorized (Supplementary Materials, Table S1) into Cluster of Orthologous Groups (COGs) classifications, and they are presented in the genome circular map in Figure 2.

The Strain ES10-3-2-2’s RAST (Rapid Annotation using Subsystem Technology) annotation genome showed 263 subsystems with 5876 coding sequences (Supplementary Materials, Figure S3).

#### 2.2.2. Radiation Resistance Genes and Pathways

The complete genome of Strain ES10-3-2-2 was annotated, and the genomic features revealed key enzymes involved in DNA recovery following ionizing radiation exposure. Additionally, the genome contains prokaryotic genes essential for repairing single-strand and double-strand DNA breaks.

The SSBR pathway in bacteria is a finely tuned system crucial for DNA maintenance and overall cell viability. Single-strand breaks, which involve the cleavage of the phosphodiester backbone of one strand of the DNA duplex, are one of the most common forms of DNA damage [19]. Repairing these breaks is critical to prevent their conversion into more deleterious double-strand breaks during DNA replication. The UvrABC excinuclease complex recognizes the structural changes caused by UV damage in DNA and repairs them by making dual incisions of 5′ and 3′ to the damaged site [20].

The genome of ES10-3-2-2 contains genes involved in radiation recovery, which plays a vital role in the included nucleotide excision repair (NER) pathway. The key enzymes are located in the genome sequence of ES10-3-2-2, which contains three copies of excinuclease ABC subunit A genes (*uvrA*; A6C57_0552, A6C57_19070, and A6C57_07285); a excinuclease ABC subunit B gene (*uvrB*; A6C57_09825); one excinuclease ABC subunit C gene (*uvrC*; A6C57_16350); the ATP-dependent DNA helicase (*uvrD*; A6C57_08045); and a DNA ligase (*LigA*; A6C57_20015). The genome also contains the transcription-coupled repair (TCR) factors, which contain key genes in the DNA-directed RNA polymerase complex (RNAP), such as *rpoA* (A6C57_11515), *rpoB* (A6C57_03695), and *rpoC* (A6C57_03690). A comparison of the genes of the NER pathways with their closely related genomes, *Deinococcus radiodurans* R1, and *Escherichia coli* K12 is provided in Table 2.

Furthermore, the inspection of the genome of Strain ES10-3-2-2 confirmed the presence of the UV damage repair endonuclease (UvdE) coding gene (uvsE; A6C57_21550) that has an 86% and 82% amino acid sequence similarity to the UVDE proteins of *Deinococcus deserti* (WP_012693877) and *D. radiodurans* (WP_034350019), respectively. *D. radiodurans* retains complete UV resistance because of the presence of this UVDE (UV damage endonuclease) repair pathway [21].

Double-strand breaks (DSBs) in DNA are severe lesions that can lead to genomic instability, mutations, and cell death if not efficiently repaired. Bacteria have evolved various DSB repair pathways to maintain genomic integrity, especially for radiation-resistant species like *D. radiodurans*. The RecFOR pathway plays a crucial role in radiation-resistant bacteria’s DNA damage repair processes. It is essential for maintaining genomic stability under extreme stress conditions, such as exposure to high radiation levels. First, the RecF protein binds to single-stranded DNA near a gap or break; second, the RecF protein binds to single-stranded DNA near a gap or break; and third, the RecO protein displaces the single-strand, DNA-binding protein and helps RecF to stabilize the DNA. RecR cooperates with RecF and RecO to facilitate the loading of RecA onto DNA to initiate repair through homologous recombination. Strain ES10-3-2-2 encodes a set of essential genes for homologous recombination, which includes recA (A6C57_1234), recF (A6C57_12250), recJ (A6C57_03175), recN (A6C57_24650), recO (A6C57_03160), recQ (A6C57_07880), recR (A6C57_19280), ssb (A6C57_13410), ruvA (A6C57_13120), ruvB (A6C57_06410), and ruvC (A6C57_18170)-encoding proteins of the recombination pathway, which play a central role in nucleic acid metabolism. A comparison of the genes of the homologous recombination (HR) pathways with their closely related genomes, *D. radiodurans* R1, and *E. coli* K12 is provided in Table 3. Similarly, the ReaA and Muts mediate pathway genes such as the DNA mismatch repair protein (MutS; A6C57_00615 and A6C57_11770), RecA protein, and the proteins implicated in the DNA repair function with the RecA and Muts genes.

#### 2.2.3. Genes Involved in Plant Growth

The beneficial effects of root-colonizing bacteria working with plants result in the increased growth and/or health of their eukaryotic hosts. Some plant-growth-promoting rhizobacteria (PGPR) exhibit various plant-beneficial features, implying that the accumulation of the related genes may have been selected in these bacteria. Plant-beneficial function contributing (PBFC) genes are distributed separately in various taxa. Table 4 gives the list of the various biosynthesis pathways between the strains.

The siderophore regulatory gene complex identified in the strain was composed of TonB; the TonB-dependent receptor (A6C57_20580, A6C57_22480); the biopolymer transport protein (ExbD, A6C57_06150); and the ExbB biopolymer transport protein (ExbB, A6C57_06155). The TonB-dependent receptors are a family of outer membrane transport proteins. TonB-dependent receptors include two copies of ExbB, one copy of ExbD, and 13 copies of TonB. The Ton complex proteins are embedded in the cytoplasmic membrane and can enter the periplasm [22]. TonB-dependent receptors detect Fe(III)-siderophore complexes on the cell’s surface [23]. Other than that, there are additional ABC transporter genes such as hmuU (A6C57_22590), the heme transport system permease protein hmuV (A6C57_22595), the heme transport system ATP-binding protein (EC:7.6.2.5) ccmC (A6C57_02225), the heme exporter protein C ccmB (A6C57_02230), the heme exporter protein B and ccmA (A6C57_25000), and the heme exporter protein A (EC:7.6.2.5). In addition, genes regarding phosphorus solubilization were also identified such as fdhA (A6C57_10225); glutathione-independent formaldehyde dehydrogenase (EC:1.2.1.46); dkgA (A6C57_12590); 2,5-diketo-D-gluconate reductase A (EC:1.1.1.346); fumC (A6C57_20830); and fumarate hydratase, class II (EC:4.2.1.2).

The genome annotation revealed genes related to plant-growth promotion, such as auxin biosynthesis genes, specifically produce indole acetic acid. The putative proteins identified by RAST related to the production of indole acetic acid were anthranilate phosphoribosyltransferase (trpD, A6C57_07705); phosphoribosyl anthranilate isomerase (trpF, A6C57_07695); the tryptophan synthase α chain (trpA, A6C57_07685); the tryptophan synthase β chain (trpB, A6C57_07690); nitrilase (A6C57_24265); and monoamine oxidase (moa, A6C57_13185). The carbon dioxide fixation gene (apaG, A6C57_17735) was also identified alongside the aforementioned. Phosphate acclimatization genes such as glucose-6-phosphate isomerase (gpi, A6C57_16520); 6-phosphofructokinase 1 (pfkA, A6C57_00700); 6-phosphofructokinase 2 (pfkB, A6C57_05450); phosphoglycerate kinase (pgk, A6C57_03080); and many more were detected in the genome of Strain ES10-3-2-2. ATPase-dependent potassium transporter genes such as potassium-transporting ATPase subunits A (kdpA, A6C57_02575); B (kdpB, A6C57_02570); C (kdpC, A6C57_02565); D (kdpD, A6C57_02555); the glutathione-regulated potassium-efflux system protein (kefB, A6C57_08505); and the glutathione-regulated potassium-efflux system ancillary protein (kefG, A6C57_08510) were also identified.

**Figure 3:**
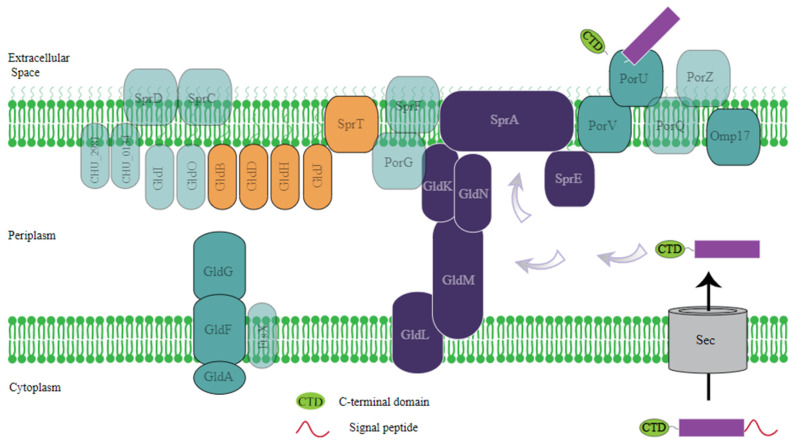
Predicted components of T9SS and the gliding motility genes used in the genome of ES10-3-2-2. The representative image illustrates the gene components associated with the Type IX secretion system (T9SS) and gliding motility, as predicted by T9GPred [26].

The genome of *Fibrivirga* spp. ES10-3-2-2 encodes several secretion systems, each serving different functional roles within the bacterial cell and its interactions with the external environment. The genome of ES10-3-2-2 has Type I, IV, and IX secretion systems, which are mainly involved in the transportation and secretion of proteins involved in bacterial adaptation. The T1SS possesses the α-hemolysin transporter, RTX toxin transporter, multiple protein transporter, adhesin protein transporter, and RaxAB-RaxC proteins. These transporters are typically used to secrete toxins and enzymes that can lyse host cells or compete with other bacteria, aiding invasion or colonization. Responsible for the secretion of RTX (repeat in toxin) family toxins, which are important for pathogenesis and defense mechanisms against other microbes. Their locus is A6C57_04240, A6C57_06760, A6C57_12130, A6C57_21585, A6C57_12910, and A6C57_24235. T6SS has core components Imp/Vas (A6C57_19575); this system functions (Supplementary Materials, Figures S4 and S5) like a molecular syringe, injecting toxic effector proteins into target cells. It plays a key role in inter-bacterial interactions. It can also target eukaryotic cells, contributing to virulence and the VgrG protein G. The needle-like structure is topped with a valine-glycine repeat protein G (VgrG) trimer that is associated with effector proteins [24]. The VgrG protein is associated with the delivery of effectors, and effectors are frequently identified based on their proximity to the encoding genes in the genome [25]. T9SS secretion system genes include porV A6C57_09150), porU (A6C57_09155), porP (A6C57_14005), porQ, and porG, and they are regulated at the transcriptional level by a signaling pathway composed of the PorXY two-component system (TCS), which contains Gld (A6C57_10305), GldB (A6C57_03925), GldD (A6C57_08085), GldF (A6C57_13325), GldG (A6C57_11530), GldH (A6C57_10940), GldL (A6C57_16335), GldM (A6C57_16340), and GldN (A6C57_16345). The OM components, PG0192/PGN_300 (annotated as an OmpH-like protein) and Omp17 (A6C57_13470), were found in the total membrane fraction. Appropriate to its 17 kDa molecular mass, the protein is called Omp17. The gliding motility genes such as SprA (A6C57_13125), SprE (A6C57_16360), and SprT (A6C57_00470) were also found. The T9SS genes in ES10-3-2-2 were also present in many other *Bacteroidetes* species [10]. A representative diagram showing the predicted T9SS and gliding motility gene components is shown in Figure 3 (Supplementary Materials, Figure S6).

### 2.3. Radiation-Resistance Analysis

The survival rate after exposure to γ and UVC irradiation was measured using early stationary phase cells (~10^7^ CFU/mL) in TGY broth (Difco, Corpus Christi, TX, USA). Since radiation resistance is a well-known feature of the genus *Deinococcus*, the survival rate of Strain ES10-3-2-2 was examined against γ and UVC radiation alongside positive and negative controls. *E. coli* K12 (KCTC 1116) served as a negative control, and *D. radiodurans* R1^T^ (DSM 20539^T^) was used as a positive control for comparison [27]. The number of colony-forming units (CFU) was determined, and the survival rate was calculated.

As a result, 76.3% and 37.7% of Strain ES10-3-2-2 survived at 4 kGy and 8 kGy of γ radiation, respectively. Additionally, *D. radiodurans* R1^T^, the positive control, saw 92.4% and 57.14% survival under the same conditions, while *E. coli* exhibited a survival rate lower than 0.001% at 4 kGy of γ radiation (Supplementary Materials, Figure S7). In this experiment, Strain ES10-3-2-2 demonstrated the characteristic shoulder in the survival curves that are typical of the γ-radiation-resistant *Deinococcus* species. Similarly, Strain ES10-3-2-2 showed resistance against UVC radiation up to 400 J/m^2^, which is lower than *D. radiodurans* R1^T^.

### 2.4. Plant-Growth Analysis

**Figure 4:**
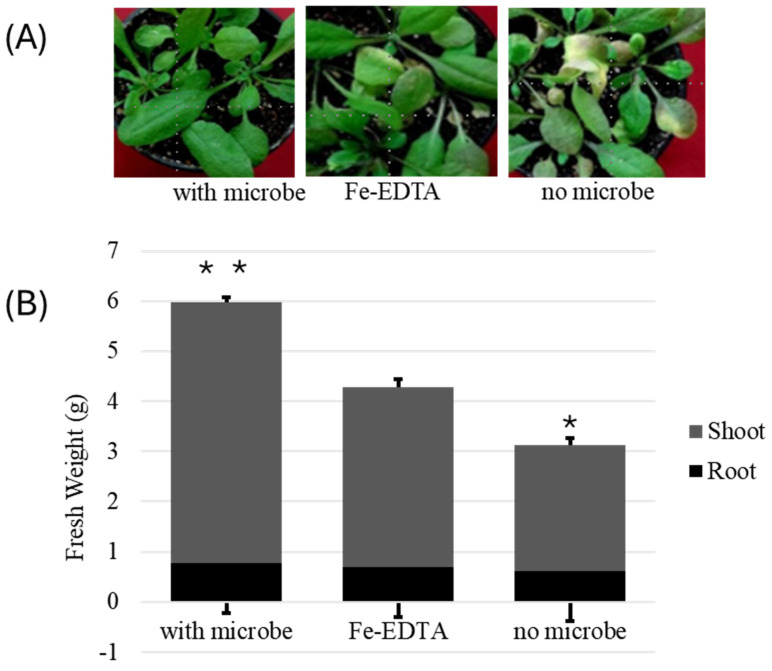
Effect of Strain ES10-3-2-2 on plant-growth parameters. (**A**) The surface of leaves detached from 20-day-old (20 DAT) grown plants. Control (no bacterial suspension), Fe-EDTA-treated plant and 2 × 10^6^ cfu/mL bacterial suspension. (**B**) Graphical representation of the fresh weights of shoots and roots at 20 DAT. The median and SE were calculated with eight plants per treatment. Significant differences between the treated plants were *, *p* < 0.02 and **, *p* < 0.05.

Plants were grown for three weeks in sterile soil with microbes and without microbes to examine the effects of Strain ES10-3-2-2 on *Arabidopsis* growth. All of the soil was first autoclaved to be void of bacteria. This was tested by serial dilutions of the autoclaved soil that streaked onto the R2A media. Plants grown in the presence of microbes (Strain ES10-3-2-2) grew healthier than plants grown without microbes and not in Fe-EDTA circumstances, regardless of the method used for soil sterilization. The bacterium-inoculated plants showed noticeably bigger leaf areas and showed no chlorosis. Strain ES10-3-2-2 treatment increased the fresh shoot rate and root weight with significant differences between treatments (*p <* 0.02 and *p <* 0.05) (Figure 4A,B). The growth promotion induced by ES10-3-2-2 remained consistent throughout all phases until the final harvest at 20 DAT (day after treatment), at which point the differences between the control group and the groups treated with ES10-3-2-2 and Fe-EDTA were more pronounced than on previous days. ES10-3-2-2 expanded the overall leaf surface higher than two-fold. Additionally, the plants cultivated in the presence of Strain ES10-3-2-2 exhibited early flowering and developed higher caudal stems with growing inflorescent plants (Supplementary Materials, Figure S8). The control plants (with no microbes) exhibited chlorosis, were more underdeveloped, and had reduced leaf sizes. The plants cultivated in sterile soil containing Fe-EDTA displayed signs of mild discoloration, elongated leaf axes, and a brittle leaf structure that was not dry (Figure 4 and Figure S8).

## 3. Discussion

In this study, Strain ES10-3-2-2, isolated from a garden soil sample in Seoul, demonstrated typical characteristics of Gram-negative, aerobic, and non-spore-forming bacteria (Figure S1). The colonies exhibited a circular shape and displayed a pale pink color on an R2A agar medium. The comprehensive genome and 16S rRNA phylogenetic analyses indicated that Strain ES10-3-2-2 should be classified within the genus *Fibrivirga*. The complete genome sequencing of Strain ES10-3-2-2 disclosed a circular chromosome of approximately 6.5 Mb, which was accompanied by three complete plasmids measuring 0.21 MB, 0.17 MB, and 81 Kb, respectively. The presence of multiple plasmids may suggest a versatile metabolic capability or adaptability, a characteristic often seen in other members. This genomic insight is crucial for the further exploration of the ecological roles and evolutionary strategies of the *Fibrivirga* Strain ES10-3-2-2.

Strain ES10-3-2-2 was subjected to 3 kGy of irradiation, significantly altering the microbial community dynamics by selecting radiation-resistant organisms [28,29]. The isolation of this strain under such conditions suggests unique genomic adaptations that confer survival advantages in extreme environments. To explore the genomic features that may be linked to its radiation resistance and ecological niche, the genome of ES10-3-2-2 was analyzed using several bioinformatics tools, including NCBI’s Prokaryotic Genome Annotation Pipeline (PGAP) [30], eggnog (v6.0) [31], and KEGG (v.110.0) [32] databases, along with T9GPred [26] specifically for identifying the components of the Type IX secretion system (T9SS). The comprehensive genomic analysis facilitated by these tools provided insights into the metabolic pathways and potential resistance mechanisms of ES10-3-2-2. PGAP helped delineate the core genomic structure, while integration with eggNOG and KEGG databases allowed for the annotation of functional capabilities and metabolic pathways, respectively. This integrative approach revealed genes that may contribute to the strain’s adaptation to irradiated environments, potentially involving DNA repair systems, the oxidative stress response, and other stress response mechanisms.

Many bacteria have developed multiple DNA repair processes to fix DNA damage and non-canonical bases, such as strand breaks, nucleotide alterations, cross-links, mismatches, and ribonucleotide incorporations, to preserve genome integrity and to guarantee cell survival. Recent developments in structural biology, genome-wide screens, and the accessibility of thousands of whole-genome sequences have sped up the identification and characterization of novel bacterial DNA repair mechanisms and new enzyme activity [33]. The DdrB protein participates in the DNA-annealing process during SSA repair, while DdrA prohibits the nucleases from breaking down DNA. A more accurate and effective ESDSA repair route can be formed by this mechanism, which also decreases the number of tiny DNA fragments and transforms them into larger pieces [34,35,36]. According to predictions made by Lim et al. [37], members of the genus *Deinococcus* are projected to possess several distinct regulatory proteins linked to the response to oxidative stress and radiation. Most bacteria trigger DNA repair genes through the RecA/Lexa-regulated SOS response to adapt to hostile conditions. LexA represses the SOS regulon genes in the absence of DNA-damaging stress. On the other hand, upon DNA damage, the activation of the protease RecA results in the inactivation of the LexA repressor, thereby causing the expression of genes that were previously silenced and initiating the recovery process of DNA damage [38]. Thus, the similarities of pathways between *Deinococcus* and ES10-3-2-2 clearly show that the pathways played a key role in resisting γ radiation.

The genomic analysis focused on the secretion systems of Strain ES10-3-2-2, a bacterium that may play roles in various environmental interactions, including plant-growth promotion and microbial competitiveness. *Fibrivirga* sp. ES10-3-2-2 features a complex genome with numerous gene-encoding components crucial for its secretion systems. These systems are instrumental for the bacterium’s ability to transport proteins and other molecules across its cellular membrane, which is pivotal for environmental adaptation and survival [39]. The Type 6 secretion system was initially identified and investigated in plant-associated *Rhizobium leguminosarum*, which influences pea nodulation and nitrogen fixation [40]. Yet, it has been shown that T6SS has a variety of roles [41], such as mediating the uptake of metal ions [42], mediating anti-bacterial [43] and anti-host activities [44], resisting environmental stress [45], inhibiting bacterial infection in the host [46], controlling the formation of bacterial biofilms [47,48], and, most recently, the function of plant-associated bacteria [49]. The Type 1 system is involved in the export of proteins that mediate interactions with plant root systems, promoting beneficial effects such as enhanced nutrient uptake. The system includes components like the α-hemolysin/cyclolysin transporter. The Type 9 system, known for the involvement of curli assembly proteins, suggests a role in the biofilm formation that takes place on plant roots, enhancing water and nutrient retention and providing a barrier against pathogens.

The current study demonstrated that plants cultivated in the presence of Strain ES10-3-2-2 that are isolated from the rhizosphere showed increased shoot growth compared to plants grown on sterile soil and Fe-EDTA. This finding coincides with previous research that revealed that plants infected with specific beneficial bacteria (PGPRs) have a higher fresh weight than those produced without microorganisms [50,51]. The enhanced shoot growth can be partly ascribed to enhanced plant nutrition (Supplementary Materials, Figure S8). Numerous agricultural studies have demonstrated the extraordinary effects of rhizobacteria in promoting plant development. In addition, the bacteria perform various unique tasks that aid in the growth and development of plants. By fine tuning and customizing a particular PGPR to the unique soil conditions of the area, its productivity can be further boosted. The presence of siderophores and auxin-associated genes, which can enhance plant growth through different mechanisms, contribute to plant nutrition and protection against phytopathogens [52,53]. In Gram-negative bacteria, the outer cell membrane transports siderophores via a combination of three transmembrane proteins such as TonB, ExbD, and ExbB [54].

Plants receive iron from siderophores made by soil microbes, which also aid in the growth of the plants. The TonB complex, which is made up of the proteins TonB, ExbB, and ExbD, facilitates this process and provides energy via the proton motive force (PMF) [54]. ATP-binding cassette (ABC) transporters within the inner membrane normally facilitate the transfer of iron-containing siderophores to the cytosol, where iron reduction occurs [55]. Direct contact between TonB and OM (outer membrane) receptors is necessary for the energy-transfer pathway in this bacterium [23,56]. Nevertheless, some fungi and bacteria that are harmful to plants are prevented from growing by bacterial siderophores. Microbes that produce siderophores provide numerous iron-chelating chemicals [57], enhancing plants’ physiological and biochemical processes in adverse environments [58]. The combined addition of Fe(III) and siderophores to the soil promotes superior plant growth compared to the addition of Fe(III) alone, as demonstrated by the observed increase in plant weight [56,59], compared to Fe-EDTA-treated plants. Many plant-growth-promoting bacteria, including *Pseudomonas*, *Rhizobium*, *Azotobacte*r, *Bacillus*, *Enterobacte*r, *Serratia*, *Azospirillum*, and *Rhizobium*, show the presence of siderophore production [59,60,61]. Therefore, Strain ES10-3-2-2 contains siderophores and auxin-related genes like other PGPR genes. Certain auxin-response-related factors may serve multiple plant functions, including reproductive and vegetative growth [62]. There is a recent discovery of an additional property of auxin in *Arabidopsis* [63,64]. These aspects include reproductive growth, callus formation and regeneration [65], reductions in biotic and abiotic stress [66], and enhanced plant root development and lateral root formation. According to Spaepen and Vanderleyden [63] and Tsavkelova et al. [67], indoles also affect the processes involved in vegetative development, initiate and enhance xylem and root development, and promote germination. The auxin-related genes of APRT, PRAI, TSa, TSb, IPAC, AO, IAR, TO, BoundA, ATFS, and N in Strain ES10-3-2-2 were also reported in the PGPRs, including *Bacillus cabrales subsp. cabrialesii TE3^T^*, *Priestia megaterium TRQ8*, and *Bacillus paralicheniformis TRQ65* [68], which improved wheat growth. Along with these genes, the potassium and phosphate assimilation gene and CO_2_ fixation genes added efficacy to growth regulation. Potassium (K) and phosphorus (P) are meant to be essential macronutrients for plant growth and development, and their availability affects crop yield [69,70]. The genome of *Fibrivirga* Strain ES10-3-2-2 has been shown to encode all the essential genes involved in radiation tolerance and plant-growth development. This strain could potentially be included in plant-growth-promoting rhizobacteria (PGPR) applications and utilized as a biofertilizer. More investigation and comprehension of the mechanisms behind PGPR-mediated phytostimulation will facilitate the development of more potent rhizobacterial strains suitable for use in various agroecological contexts.

## 4. Materials and Methods

### 4.1. Isolation and Growth

A rhizosphere soil sample from a garden in Nowon-gu, Seoul, Korea (GPS: N 37°37′41″, E 127°05′25″) yielded Strain ES10-3-2-2. Using a cobalt-60 γ irradiator, a soil sample was exposed to 3 kGy of radiation, which resulted in isolating γ-ray-resistant colonies. In summary, as previously reported [71,72], a soil sample exposed to radiation from γ rays was serially diluted and plated on R2A agar (Difco, Franklin Lakes, NJ, USA). A pink-to-red-colored bacterial strain was identified and purified after plates were cultured at 25 °C for seven days. The isolated strain was designated as Strain ES10-3-2-2. The isolate was preserved in R2A broth containing 20% (*w*/*v*) glycerol and stored at −80 °C. The pure culture of Strain ES10-3-2-2 was deposited at the NITE Biological Resource Center (NBRC) under the accession number NBRC 115440.

### 4.2. Genome Sequencing and Phylogenomic Analysis

According to the manufacturer’s instructions, genomic DNA from Strain ES10-3-2-2 was isolated and purified using the DNeasy PowerLyzer Microbial Kit (Qiagen, Ann Arbor, MI, USA). The genome was sequenced using Pacific Biosciences RS II technologies. The genomic library was constructed following the Pacific Biosciences RS II sequencing technology method. A total of 237,513 sequencing reads were obtained and assembled using the hierarchical genome assembly process (HGAP) v.3.0 with the default settings. ContEst16S was used to measure the quality of the assembly [73]. A phylogenetic tree based on the genome and 16S rRNA was constructed using the Type (Strain) Genome Server (TYGS) [74] employing default settings.

### 4.3. Genome Annotation

The whole-genome sequences of the strains were deposited in the GenBank (www.ncbi.nlm.nih.gov/) database and annotated using the National Center for Biotechnology Information, Prokaryotic Genome Annotation Pipeline (PGAP) [30]. BLASTp (v.2.15.0) was used to analyze the protein similarity [75] between Strain ES10-3-2-2, *D. radiodurans* R1^T^, and *E. coli* K12. The open reading frame was predicted using Prodigal (v.2.6.3) [76] and annotated at the RAST server (https://rast.nmpdr.org/rast.cgi, accessed on 5 April 2024) [77,78,79].

### 4.4. Radiation Resistant Analysis

#### 4.4.1. γ Irradiation Analysis

To determine the potential of the γ-ray and UVC irradiation resistance, exponentially grown cells (~10^8^ CFU/mL) were taken and exposed to radiation at room temperature using a cobalt-60 γ-ray device (point source; AECL, IR-79) [80,81]. The actual doses were within 2% of the goal dose, and the γ-ray strength was roughly 100 kCi at a dose rate of 70 Gy min^−1^. Strain ES10-3-2-2 and two control strains were serially diluted in 0.85% NaCl after radiation and then spread out on R2A plates, respectively. The plates were incubated at 25 °C for three days until visible colonies developed; at this point, their numbers were counted. As a positive control, *D. radiodurans* R1^T^ (=DSM 20539^T^) was employed, and *E. coli* K12 (=KCTC 1116) was employed as a negative control.

#### 4.4.2. UVC Irradiation Analysis

For the analysis of UVC radiation resistance, cells were cultured in R2A broth (Difco) to an early stationary phase and then adjusted to approximately 10^8^ CFU/mL [81,82]. These cells were serially diluted in a 0.85% NaCl solution and plated on R2A agar. Plates were exposed to UVC radiation at room temperature using a UVC ultraviolet crosslinker (UVP, CX-2000, Upland, CA, USA) set at a 254 nm wavelength. The radiation dose rate was set at 20 J/m^2^/s, with varying radiation doses obtained by modifying the exposure duration. Following radiation exposure, the plates were incubated at 30 °C for two days before colony counts were performed.

### 4.5. Plant Growth Analysis

#### Plant Cultivation and Treatments

The wild-type *Arabidopsis thaliana* seed ecotype Col-0 was first washed several times with sterile water, surface-sterilized in 1 mL of 70% (*v*/*v*) ethanol for 1 min, and then treated with 1 mL of bleach solution [50% (*v*/*v*) bleach/0.05% (*v*/*v*) Tween-20] for 5 min with vortexing. Germinated seeds were grown for two weeks in a plant development chamber (23 ± 2 °C, 12 h photoperiod, and 70–80% relative humidity). Bacteria were cultured in 50 mL of an R2A broth at 28 °C for 24 h while being shaken (at 250 rpm) to prepare the inoculum. After a 5 min centrifugation at 6000× *g*, the bacterial cells were cleaned with sterile distilled water, resuspended in 10 mL of a sterile saline solution, and utilized for priming and inoculation (at a concentration of 10^8^ cfu/mL). Following two weeks of growth, individual plants were root-primed by air drying and immersion in a bacterial suspension for one hour. Following root priming, every plant was moved into a brand-new pot (5 × 7 cm) filled with a coco-peat mixture. The roots of each plant were then inoculated with 1 mL of a bacterial solution. Ten plants per treatment and 4 plants per pot were kept. The plants were maintained under the same circumstances in a plant-growth chamber and were collected three weeks after the inoculation. Three treatments were designed for examination: (1) sterilized compost soil, (2) a sterile solution of Fe-EDTA (13 μM), and (3) microbial inoculum (which served as a source for Strain ES10-3-2-2). A statistical data analysis was performed in between the duplicates. After harvesting, the leaf area (mm^2^) of the individual leaves in all of the treatments was calculated. The fresh shoot and root weight (mg) were also measured. The plants were photographed at regular intervals and on the day of harvesting.

### 4.6. Statistical Analysis

All of the data of the *Arabidopsis* growth parameters were analyzed by one-way ANOVA followed by Tukey’s test, with the bacterial inoculation method considered as the independent variable. Using SPSS software (v.26), all statistical analyses were computed at the significance level of *p* < 0.05. Microsoft Excel 2018 was used to compute the mean and standard deviation for the trials between duplicates.

## 5. Conclusions

The complete genome analysis provided insight into the genetic features that support the studied strain’s plant-associated lifestyle and habitat adaptation, such as radiation resistance. The understanding of cellular resilience and adaptation has been broadened by investigating these radiation-resistant microbes. Studying extremophiles is important for many fields, including biotechnology, where their resilience mechanisms are used to create new applications, as well as astrobiology, where they simulate the possibility of life on other radioactively altered celestial worlds. It was also clarified that the presence of genes involved in plant growth impacted the outcomes of the in vitro experiment. Therefore, mocking the genes responsible for plant growth might give detailed illustrations of the importance of the bacteria. Therefore, further fieldwork-controlled research and short- and long-term analyses are required to fully comprehend how individual plants, populations, and plant communities respond to this interaction. The secretion systems of *Fibrivirga* sp. ES10-3-2-2, particularly Types I, VI, and IX, highlight the bacterium’s potential as a beneficial organism in agriculture. These systems support the bacterium’s role in promoting plant growth, potentially making it useful in sustainable agricultural practices. The knowledge gained from this investigation provides tactical direction for strain improvement in the future and provides a solid basis for the bacterium’s possible application in farming methods. The isolated bacterium investigated in this study, belonging to the genus *Fibrivirga*, demonstrated remarkable γ radiation tolerance resistance and exhibited characteristics that promote plant growth. Such dual functionality is rare and of high interest as it suggests potential applications in enhancing crop resilience to radiative environmental conditions.

## Figures and Tables

**Table 1:** Summary of the genome-sequencing project for Strain ES10-3-2-2. This table provides a comprehensive overview of the genomic characteristics of Strain ES10-3-2-2, as sequenced across its chromosome and three plasmids.

Features	Chromosome	Plasmid 1	Plasmid 2	Plasmid 3
Strain	ES10-3-2-2	ES10-3-2-2	ES10-3-2-2	ES10-3-2-2
Number of contigs	1	1	1	1
GenBank Accession No.	CP015317	CP155472	CP155473	CP155474
Chromosome size (pb)	6,408,035	212,574	175,683	81,564
GC content	52.8	52.4	49.0	52.9
Sequencing coverage	71	71	71	71
Genes	5252	189	159	91
rRNA	9	0	0	0
5s rRNAs	3	0	0	0
16S rRNAs	3	0	0	0
23S rRNA	3	0	0	0
tRNAs	43	0	0	0
ncRNAs	2	0	0	0
Pseudo genes	45	0	0	0

**Table 2:** The presence of DNA repair genes involved in the excision repair pathways compared with *D. radiodurans* R1 and *E. coli* K12.

S. No.	Length	Function	Sequence Similarity (BLAST)	
			*D. radiodurans* R1	*E. coli* K12
1	999	Excinuclease ABC subunit A paralog	39.2	44.2
2	977	Excinuclease ABC subunit A	51.8	51.9
3	767	ATP-dependent DNA helicase UvrD/PcrA	37.5	37.2
4	674	Excinuclease ABC subunit B	50.0	54.8
5	605	Excinuclease ABC subunit C	31.1	33.4
6	845	Excinuclease ABC subunit A paralog	71.3	40.3
7	690	DNA ligase (NAD (+))	34.4	40.6
8	886	Excinuclease ABC subunit A paralog of unknown function	63.5	38.0

**Table 3:** The presence of DNA repair genes involved in the homologous recombination pathways compared with *D. radiodurans* R1 and *E. coli* K12.

S. No.	Length	Function	Sequence Similarity (BLAST)	
			*D. radiodurans* R1	*E. coli* K12
1	703	ATP-dependent DNA helicase RecG	41.1	40.0
2	193	Crossover junction endodeoxyribonuclease RuvC	38.6	36.8
3	365	DNA recombination and repair protein RecF	26.3	26.4
4	231	DNA recombination and repair protein RecO	0.0	0.0
5	459	DNA repair protein RadA	42.9	46.0
6	555	DNA repair protein RecN	30.7	29.8
7	1029	Exonuclease SbcC	34.8	50.0
8	418	Exonuclease SbcD	32.3	32.5
9	836	Helicase PriA essential for oriC/DnaA	36.6	37.6
10	196	Holliday junction ATP-dependent DNA helicase RuvA	33.5	39.7
11	343	Holliday junction ATP-dependent DNA helicase RuvB	54.4	59.3
12	363	RecA protein	59.2	59.8
13	206	Recombination protein RecR	45.8	40.4
14	590	Single-stranded, DNA-specific exonuclease RecJ	32.2	31.6

**Table 4:** Lists of various biosynthesis pathways involved in plant-growth-promoting activity across Strain ES10-3-2-2 and its closely related genomes. The analysis was based on KEGG pathways (ECs). Each row shows a pathway and its corresponding total ECs, with subsequent columns displaying the count and percentage of ECs identified in each strain.

KEGG Map	Distinct ECs	Strain ES10-3-2-2	“*F.* *algicola* JA-25”	*Fibrella* *aestuarina* BUZ 2^T^	*Spirosoma* *linguale* SM 74^T^
Biosynthesis of plant hormones	131	66 (50.4%)	66 (50.4%)	76 (58.0%)	75 (57.3%)
Brassinosteroid biosynthesis	3	1 (33.3%)	1 (33.3%)	2 (66.7%)	2 (66.7%)
Fatty acid biosynthesis	21	10 (47.6%)	10 (47.6%)	10 (47.6%)	12 (57.1%)
Terpenoid backbone biosynthesis	27	10 (37.0%)	10 (37.0%)	15 (55.6%)	14 (51.9%)
Zeatin biosynthesis	9	0	0	0	0

## Data Availability

The data used in this study are available under the NCBI accession numbers CP015317, CP155472, CP155473, and CP155474.

## Supplementary Materials

The following supporting information can be downloaded at https://www.mdpi.com/article/10.3390/genes15081048/s1, Table S1: COG analysis of the genome ES10-3-2-2. Figure S1: Transmission electron micrographs of Strain ES10-3-2-2. Figure S2: 16S rRNA-based phylogenetic tree of ES10-3-2-2 and related type strains. Figure S3: Subsystem feature of Strain ES10-3-2-2 revealed by RAST server. Figure S4: Predicted T6SS gene cluster of ES10-3-2-2. Figure S5: Gene cluster visualization of Type 6 secretion system and gliding motility genes. Figure S6: Gene cluster visualization of Type 9 secretion system genes. Figure S7: Radiation and representative survival curve of Strain ES10-3-2-2. Figure S8: Plant growth shows the effect of inoculum.

## References

[1] Trivedi P., Leach J.E., Tringe S.G., Sa T., Singh B.K. (2020). Plant–microbiome interactions: From community assembly to plant health. Nat. Rev. Microbiol..

[2] Olanrewaju O.S., Babalola O.O. (2022). The rhizosphere microbial complex in plant health: A review of interaction dynamics. J. Integr. Agric..

[3] Adomako M.O., Roiloa S., Yu F.-H. (2022). Potential roles of soil microorganisms in regulating the effect of soil nutrient heterogeneity on plant performance. Microorganisms.

[4] Kabato W.S., Janda T., Molnár Z. (2023). Unveiling the significance of rhizosphere: Implications for plant growth, stress response, and sustainable agriculture. Plant Physiol. Biochem..

[5] Omae N., Tsuda K. (2022). Plant-Microbiota Interactions in Abiotic Stress Environments. Mol. Plant-Microbe Interact..

[6] L’Annunziata M.F. (2007). Introduction: Radioactivity and our well-being. Radioactivity.

[7] Daly M.J. (2009). A new perspective on radiation resistance based on *Deinococcus radiodurans*. Nat. Rev. Microbiol..

[8] Ibáñez A., Garrido-Chamorro S., Barreiro C. (2023). Microorganisms and Climate Change: A Not So Invisible Effect. Microbiol. Res..

[9] Filloux A. (2022). Bacterial protein secretion systems: Game of types. Microbiology.

[10] Lasica A.M., Ksiazek M., Madej M., Potempa J. (2017). The type IX secretion system (T9SS): Highlights and recent insights into its structure and function. Front. Cell. Infect. Microbiol..

[11] Krisko A., Radman M. (2013). Biology of extreme radiation resistance: The way of *Deinococcus radiodurans*. Cold Spring Harb. Perspect. Biol..

[12] Megali L., Glauser G., Rasmann S. (2014). Fertilization with beneficial microorganisms decreases tomato defenses against insect pests. Agron. Sustain. Dev..

[13] Nazir N., Kamili A., Shah D. (2019). Mechanism of Plant Growth Promoting Rhizobacteria (PGPR) in enhancing plant growth—A Review. Int. J. Manag. Technol. Eng..

[14] Neilands J.B. (1981). Microbial iron compounds. Annu. Rev. Biochem..

[15] García-López M., Meier-Kolthoff J.P., Tindall B.J., Gronow S., Woyke T., Kyrpides N.C., Hahnke R.L., Göker M. (2019). Analysis of 1,000 type-strain genomes improves taxonomic classification of Bacteroidetes. Front. Microbiol..

[16] Park S., Cho J.Y., Jung D.-H., Jang S.W., Eom J.H., Nam S.W., Kwon D.R., Ryu J., Kim K.T. (2022). *Fibrivirga algicola* gen. nov., sp. nov., an algicidal bacterium isolated from a freshwater river. Antonie Van Leeuwenhoek.

[17] Lefort V., Desper R. (2015). FastME OG. 2.0: A comprehensive, accurate, and fast distance-based phylogeny inference program. Mol. Biol. Evol..

[18] Farris J.S. (1972). Estimating phylogenetic trees from distance matrices. Am. Nat..

[19] Chatterjee N., Walker G.C. (2017). Mechanisms of DNA damage, repair, and mutagenesis. Environ. Mol. Mutagen.

[20] Petit C., Sancar A. (1999). Nucleotide excision repair: From *E. coli* to man. Biochimie.

[21] Earl A.M., Rankin S.K., Kim K.-P., Lamendola O.N., Battista J.R. (2002). Genetic evidence that the uvsE gene product of *Deinococcus radiodurans* R1 is a UV damage endonuclease. J. Bacteriol..

[22] Kampfenkel K., Braun V. (1992). Membrane topology of the *Escherichia coli* ExbD protein. J. Bacteriol..

[23] Krewulak K.D., Vogel H.J. (2008). Structural biology of bacterial iron uptake. Biochim. Biophys. Acta (BBA)-Biomembr..

[24] Ho B.T., Dong T.G., Mekalanos J.J. (2014). A view to a kill: The bacterial type VI secretion system. Cell Host Microbe.

[25] Spiewak H.L., Shastri S., Zhang L., Schwager S., Eberl L., Vergunst A.C., Thomas M.S. (2019). Burkholderia cenocepacia utilizes a type VI secretion system for bacterial competition. Microbiologyopen.

[26] Sahoo A.K., Vivek-Ananth R.P., Chivukula N., Rajaram S.V., Mohanraj K., Khare D., Acharya C., Samal A. (2023). T9GPred: A Comprehensive Computational Tool for the Prediction of Type 9 Secretion System, Gliding Motility, and the Associated Secreted Proteins. ACS Omega.

[27] Kämpfer P., Lodders N., Huber B., Falsen E., Busse H.-J. (2008). *Deinococcus aquatilis* sp. nov., isolated from water. Int. J. Syst. Evol. Microbiol..

[28] Ogwu M.C., Srinivasan S., Dong K., Ramasamy D., Waldman B., Adams J.M. (2019). Community Ecology of Deinococcus in Irradiated Soil. Microb. Ecol..

[29] Ogwu M.C., Kerfahi D., Song H., Dong K., Seo H., Lim S., Srinivasan S., Kim M.K., Waldman B., Adams J.M. (2019). Changes in soil taxonomic and functional diversity resulting fromgamma irradiation. Sci. Rep..

[30] Tatusova T., DiCuccio M., Badretdin A., Chetvernin V., Nawrocki E.P., Zaslavsky L., Lomsadze A., Pruitt K.D., Borodovsky M., Ostell J. (2016). NCBI prokaryotic genome annotation pipeline. Nucleic Acids Res..

[31] Hernández-Plaza A., Szklarczyk D., Botas J., Cantalapiedra C.P., Giner-Lamia J., Mende D.R., Kirsch R., Rattei T., Letunic I., Jensen L.J. (2023). eggNOG 6.0: Enabling comparative genomics across 12 535 organisms. Nucleic Acids Res..

[32] Kanehisa M., Furumichi M., Sato Y., Kawashima M., Ishiguro-Watanabe M. (2023). KEGG for taxonomy-based analysis of pathways and genomes. Nucleic Acids Res..

[33] Wozniak K.J., Simmons L.A. (2022). Bacterial DNA excision repair pathways. Nat. Rev. Microbiol..

[34] Harris D.R., Tanaka M., Saveliev S.V., Jolivet E., Earl A.M., Cox M.M., Battista J.R. (2004). Preserving genome integrity: The DdrA protein of *Deinococcus radiodurans* R1. PLoS Biol..

[35] Xu G., Lu H., Wang L., Chen H., Xu Z., Hu Y., Tian B., Hua Y. (2010). DdrB stimulates single-stranded DNA annealing and facilitates RecA-independent DNA repair in *Deinococcus radiodurans*. DNA Repair.

[36] Basu B., Apte S.K. (2012). Gamma radiation-induced proteome of *Deinococcus radiodurans* primarily targets DNA repair and oxidative stress alleviation. Mol. Cell. Proteom..

[37] Lim S., Jung J.-H., Blanchard L., de Groot A. (2019). Conservation and diversity of radiation and oxidative stress resistance mechanisms in Deinococcus species. FEMS Microbiol. Rev..

[38] Erill I., Campoy S., Barbé J. (2007). Aeons of distress: An evolutionary perspective on the bacterial SOS response. FEMS Microbiol. Rev..

[39] Yin R., Cheng J., Lin J. (2024). The role of the type VI secretion system in the stress resistance of plant-associated bacteria. Stress Biol..

[40] Bladergroen M.R., Badelt K., Spaink H. (2003). Infection-blocking genes of a symbiotic *Rhizobium leguminosarum* strain that are involved in temperature-dependent protein secretion. Mol. Plant-Microbe Interact..

[41] Lin J., Xu L., Yang J., Wang Z., Shen X. (2021). Beyond dueling: Roles of the type VI secretion system in microbiome modulation, pathogenesis and stress resistance. Stress Biol..

[42] Han Y., Wang T., Chen G., Pu Q., Liu Q., Zhang Y., Xu L., Wu M., Liang H. (2019). A Pseudomonas aeruginosa type VI secretion system regulated by CueR facilitates copper acquisition. PLoS Pathog..

[43] Trunk K., Peltier J., Liu Y.-C., Dill B.D., Walker L., Gow N.A., Stark M.J., Quinn J., Strahl H., Trost M. (2018). The type VI secretion system deploys antifungal effectors against microbial competitors. Nat. Microbiol..

[44] Coulthurst S. (2019). The Type VI secretion system: A versatile bacterial weapon. Microbiology.

[45] Wan B., Zhang Q., Ni J., Li S., Wen D., Li J., Xiao H., He P., Ou H.-Y., Tao J. (2017). Type VI secretion system contributes to Enterohemorrhagic *Escherichia coli* virulence by secreting catalase against host reactive oxygen species (ROS). PLoS Pathog..

[46] Bendor L., Weyrich L.S., Linz B., Rolin O.Y., Taylor D.L., Goodfield L.L., Smallridge W.E., Kennett M.J., Harvill E.T. (2015). Type six secretion system of *Bordetella bronchiseptica* and adaptive immune components limit intracellular survival during infection. PLoS ONE.

[47] Lin J., Cheng J., Chen K., Guo C., Zhang W., Yang X., Ding W., Ma L., Wang Y., Shen X. (2015). The icmF3 locus is involved in multiple adaptation-and virulence-related characteristics in *Pseudomonas aeruginosa* PAO1. Front. Cell. Infect. Microbiol..

[48] Chen L., Zou Y., Kronfl A.A., Wu Y. (2020). Type VI secretion system of *Pseudomonas aeruginosa* is associated with biofilm formation but not environmental adaptation. Microbiologyopen.

[49] Wang J., Yin R., Yang J., Cheng J., Lin J. (2023). Research progress in the function of type VI secretion system in plant-associated bacteria. Acta Microbiol. Sin..

[50] Persello-Cartieaux F., David P., Sarrobert C., Thibaud M.-C., Achouak W., Robaglia C., Nussaume L. (2001). Utilization of mutants to analyze the interaction between *Arabidopsis thaliana* and its naturally root-associated Pseudomonas. Planta.

[51] Ryu C.-M., Hu C.-H., Locy R.D., Kloepper J.W. (2005). Study of mechanisms for plant growth promotion elicited by rhizobacteria in *Arabidopsis thaliana*. Plant Soil..

[52] Scavino A.F., Pedraza R.O. (2013). The role of siderophores in plant growth-promoting bacteria. Bacteria in Agrobiology: Crop Productivity.

[53] Zhang H., Zhu J., Gong Z., Zhu J.-K. (2022). Abiotic stress responses in plants. Nat. Rev. Genet..

[54] Khasheii B., Mahmoodi P., Mohammadzadeh A. (2021). Siderophores: Importance in bacterial pathogenesis and applications in medicine and industry. Microbiol. Res..

[55] Guillon L., Altenburger S., Graumann P.L., Schalk I.J. (2013). Deciphering protein dynamics of the siderophore pyoverdine pathway in *Pseudomonas aeruginosa*. PLoS ONE.

[56] Garcia-Herrero A., Peacock R.S., Howard S.P., Vogel H.J. (2007). The solution structure of the periplasmic domain of the TonB system ExbD protein reveals an unexpected structural homology with siderophore-binding proteins. Mol. Microbiol..

[57] Alam A. (2014). Soil degradation: A challenge to sustainable agriculture. Int. J. Sci. Res. Agric. Sci..

[58] Sultana S., Alam S., Karim M.M. (2021). Screening of siderophore-producing salt-tolerant rhizobacteria suitable for supporting plant growth in saline soils with iron limitation. J. Agric. Food Res..

[59] De Serrano L.O. (2017). Biotechnology of siderophores in high-impact scientific fields. Biomol. Concepts.

[60] Pahari A., Mishra B. (2017). Characterization of siderophore producing rhizobacteria and its effect on growth performance of different vegetables. Int. J. Curr. Microbiol. Appl. Sci..

[61] Loper J.E., Henkels M.D. (1999). Utilization of heterologous siderophores enhances levels of iron available to *Pseudomonas putida* in the rhizosphere. Appl. Environ. Microbiol..

[62] Zhang Q., Gong M., Xu X., Li H., Deng W. (2022). Roles of auxin in the growth, development, and stress tolerance of horticultural plants. Cells.

[63] Spaepen S., Vanderleyden J. (2011). Auxin and plant-microbe interactions. Cold Spring Harb. Perspect. Biol..

[64] Xu J., Han L., Xia S., Zhu R., Kang E., Shang Z. (2023). ATANN3 is involved in extracellular ATP-regulated auxin distribution in *Arabidopsis thaliana* seedlings. Plants.

[65] Kazan K. (2013). Auxin and the integration of environmental signals into plant root development. Ann. Bot..

[66] Egamberdieva D. (2009). Alleviation of salt stress by plant growth regulators and IAA producing bacteria in wheat. Acta Physiol. Plant..

[67] Tsavkelova E., Klimova S.Y., Cherdyntseva T., Netrusov A. (2006). Hormones and hormone-like substances of microorganisms: A review. Appl. Biochem. Microbiol..

[68] Zepeda M.A., Ruiz V.V., Cota F.I.P., Chinchilla-Soto C., de la Cruz Torres E., Ibba M.I., Alvarado M.I.E., de los Santos Villalobos S. (2024). Genomic insights of a native bacterial consortium for wheat production sustainability. Curr. Res. Microb. Sci..

[69] Malhotra H., Vandana Sharma S., Pandey R. (2018). Phosphorus nutrition: Plant growth in response to deficiency and excess. Plant Nutrients and Abiotic Stress Tolerance.

[70] Wang Y., Li W., Du B., Li H. (2021). Effect of biochar applied with plant growth-promoting rhizobacteria (PGPR) on soil microbial community composition and nitrogen utilization in tomato. Pedosphere.

[71] Cho C., Lee D., Jeong D., Kim S., Kim M.K., Srinivasan S. (2023). Characterization of radiation-resistance mechanism in *Spirosoma montaniterrae* DY10T in terms of transcriptional regulatory system. Sci. Rep..

[72] Lee J.-J., Srinivasan S., Lim S., Joe M., Lee S.H., Kwon S.A., Kwon Y.J., Lee J., Choi J.J., Lee H.M. (2014). *Hymenobacter swuensis* sp. nov., a Gamma-Radiation-Resistant Bacteria Isolated from Mountain Soil. Curr. Microbiol..

[73] Lee I., Chalita M., Ha S.-M., Na S.-I., Yoon S.-H., Chun J. (2017). ContEst16S: An algorithm that identifies contaminated prokaryotic genomes using 16S RNA gene sequences. Int. J. Syst. Evol. Microbiol..

[74] Meier-Kolthoff J.P., Göker M. (2019). TYGS is an automated high-throughput platform for state-of-the-art genome-based taxonomy. Nat. Commun..

[75] Camacho C., Coulouris G., Avagyan V., Ma N., Papadopoulos J., Bealer K., Madden T.L. (2009). BLAST+: Architecture and applications. BMC Bioinform..

[76] Hyatt D., Chen G.-L., LoCascio P.F., Land M.L., Larimer F.W., Hauser L.J. (2010). Prodigal: Prokaryotic gene recognition and translation initiation site identification. BMC Bioinform..

[77] Aziz R.K., Bartels D., Best A.A., DeJongh M., Disz T., Edwards R.A., Formsma K., Gerdes S., Glass E.M., Kubal M. (2008). The RAST Server: Rapid annotations using subsystems technology. BMC Genom..

[78] Overbeek R., Olson R., Pusch G.D., Olsen G.J., Davis J.J., Disz T., Edwards R.A., Gerdes S., Parrello B., Shukla M. (2014). The SEED and the Rapid Annotation of microbial genomes using Subsystems Technology (RAST). Nucleic Acids Res..

[79] Brettin T., Davis J.J., Disz T., Edwards R.A., Gerdes S., Olsen G.J., Olson R., Overbeek R., Parrello B., Pusch G.D. (2015). RASTtk: A modular and extensible implementation of the RAST algorithm for building custom annotation pipelines and annotating batches of genomes. Sci. Rep..

[80] Kim J.-Y., Kim D.-U., Kang M.-S., Jang J.H., Kim S.J., Kim M.J., Lee J.Y., Lee Y.S., Zhang J., Lim S. (2018). *Roseomonas radiodurans* sp. nov., a gamma-radiation-resistant bacterium isolated from gamma ray-irradiated soil. Int. J. Syst. Evol. Microbiol..

[81] Im S., Song D., Joe M., Kim D., Park D.-H., Lim S. (2013). Comparative survival analysis of 12 histidine kinase mutants of *Deinococcus radiodurans* after exposure to DNA-damaging agents. Bioprocess. Biosyst. Eng..

[82] Selvam K., Duncan J.R., Tanaka M., Battista J.R. (2013). DdrA, DdrD, and PprA: Components of UV and mitomycin C resistance in *Deinococcus radiodurans* R1. PLoS ONE.

