# Supplementary material for: Radiation-Tolerant Fibrivirga spp. from Rhizosphere Soil: Genome Insights and Potential in Agriculture
Source: Genes (Basel). 2024 Aug 9;15(8):1048. doi: 10.3390/genes15081048 (PMC11354047; doi:10.3390/genes15081048)
Supplement: Supplementary file 1 [file genes-15-01048-s001.zip › Supp. Figure S2.pptx]

## Slide 1
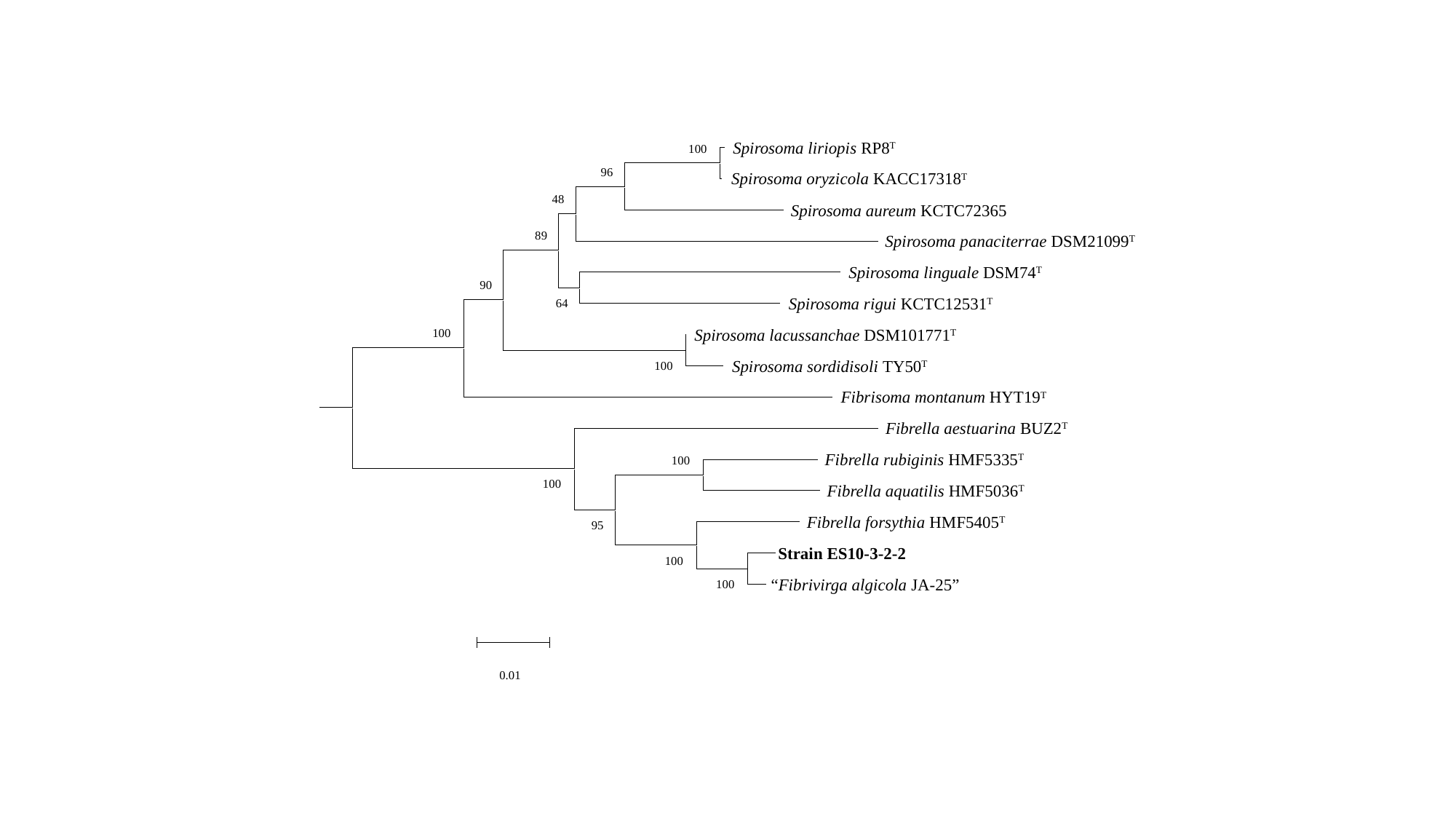

Spirosoma liriopis RP8T
100
96
 Spirosoma oryzicola KACC17318T
48
 Spirosoma aureum KCTC72365
89
 Spirosoma panaciterrae DSM21099T
 Spirosoma linguale DSM74T
90
 Spirosoma rigui KCTC12531T
64
 Spirosoma lacussanchae DSM101771T
100
 Spirosoma sordidisoli TY50T
100
 Fibrisoma montanum HYT19T
 Fibrella aestuarina BUZ2T
 Fibrella rubiginis HMF5335T
100
100
 Fibrella aquatilis HMF5036T
 Fibrella forsythia HMF5405T
95
Strain ES10-3-2-2
100
“Fibrivirga algicola JA-25”
100
0.01
