# Supplementary material for: Radiation-Tolerant Fibrivirga spp. from Rhizosphere Soil: Genome Insights and Potential in Agriculture
Source: Genes (Basel). 2024 Aug 9;15(8):1048. doi: 10.3390/genes15081048 (PMC11354047; doi:10.3390/genes15081048)
Supplement: Supplementary file 1 [file genes-15-01048-s001.zip › Supp. Figure S7 Radiation.pptx]

## Slide 1
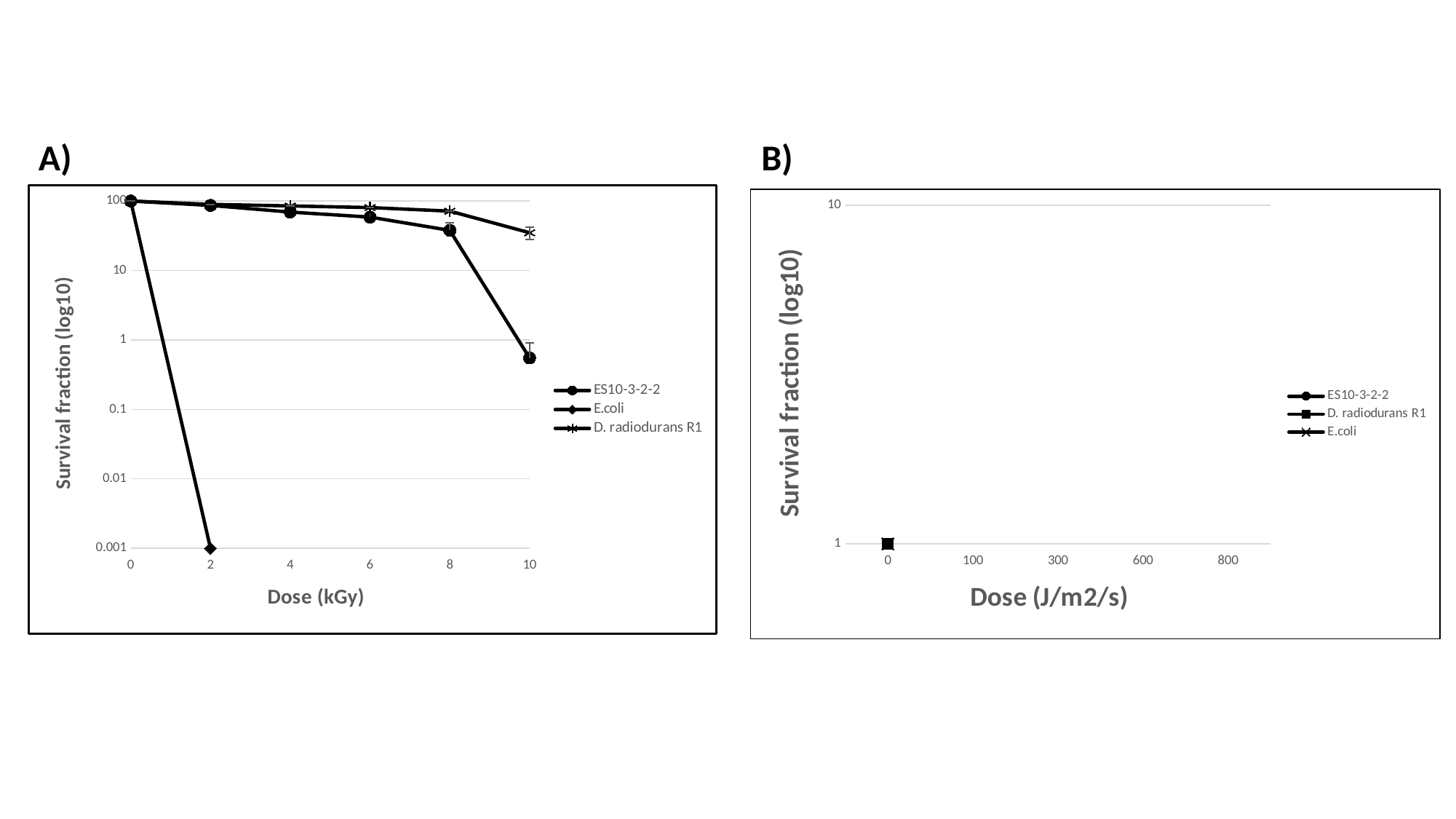

B)
A)
### Chart
| Category | ES10-3-2-2 | E.coli | D. radiodurans R1 |
|---|---|---|---|
| 0 | 100.0 | 100.0 | 100.0 |
| 2 | 86.5079365079365 | 0.001 | 89.5 |
| 4 | 69.3204365079365 | None | 85.0 |
| 6 | 58.730158730158735 | None | 80.5 |
| 8 | 37.79761904761905 | None | 71.5 |
| 10 | 0.5505952380952381 | None | 35.0 |
### Chart
| Category | ES10-3-2-2 | D. radiodurans R1 | E.coli |
|---|---|---|---|
| 0 | 1.0 | 1.0 | 1.0 |
| 100 | 0.7857142857142857 | 0.7638888888888888 | 1e-06 |
| 300 | 0.5357142857142857 | 0.028541666666666667 | 0.0 |
| 600 | 0.4107142857142857 | 0.0005833333333333333 | 0.0 |
| 800 | 0.3392857142857143 | 4.652777777777778e-05 | 0.0 |
