# Supplementary figures and images for: Radiation-Tolerant Fibrivirga spp. from Rhizosphere Soil: Genome Insights and Potential in Agriculture
Source: Genes (Basel). 2024 Aug 9;15(8):1048. doi: 10.3390/genes15081048 (PMC11354047; doi:10.3390/genes15081048)

## Slide 1
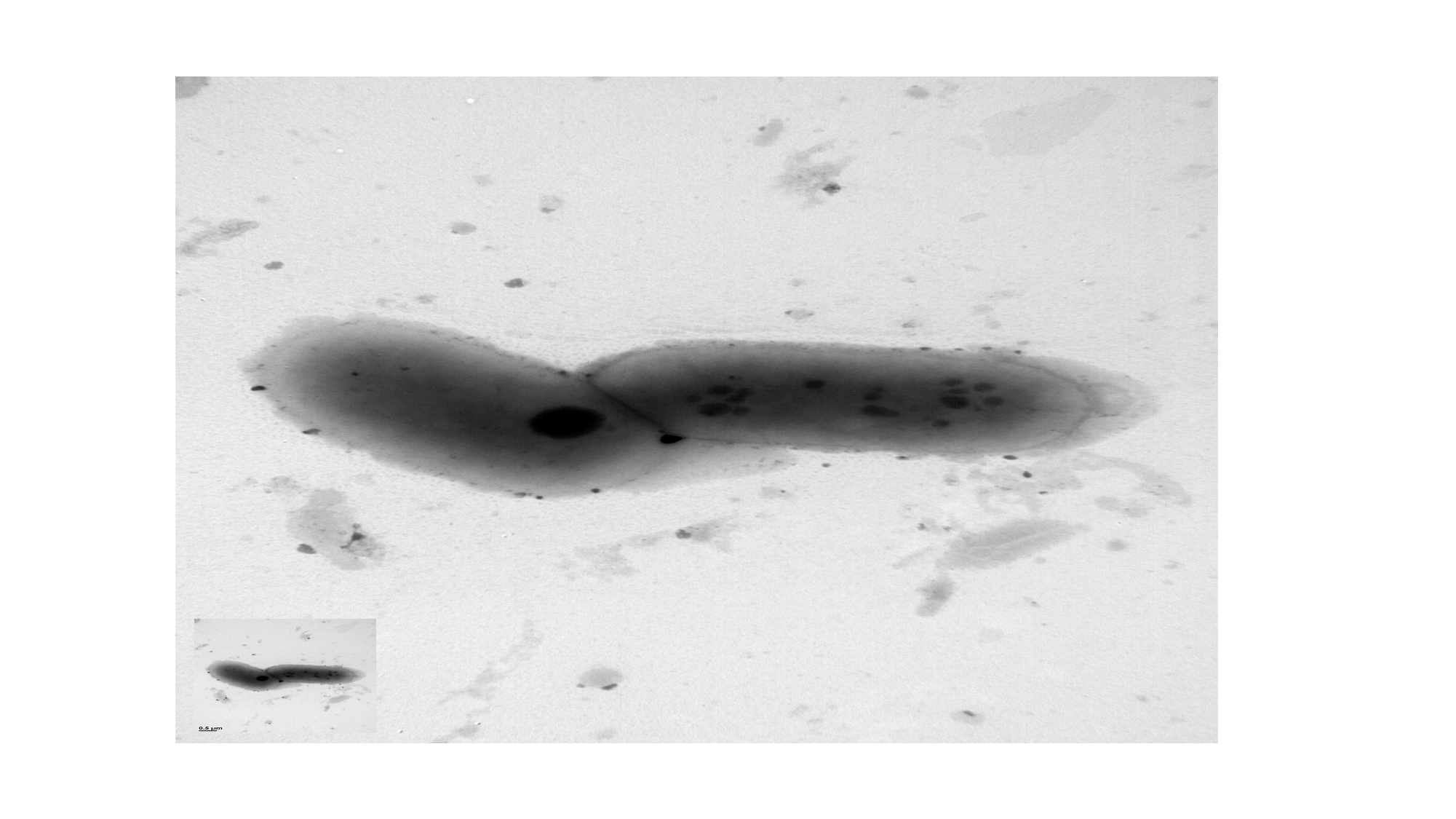

Supplement: Supplementary file 1 [file genes-15-01048-s001.zip › Supp. Figure S1.pptx]

## Slide 1
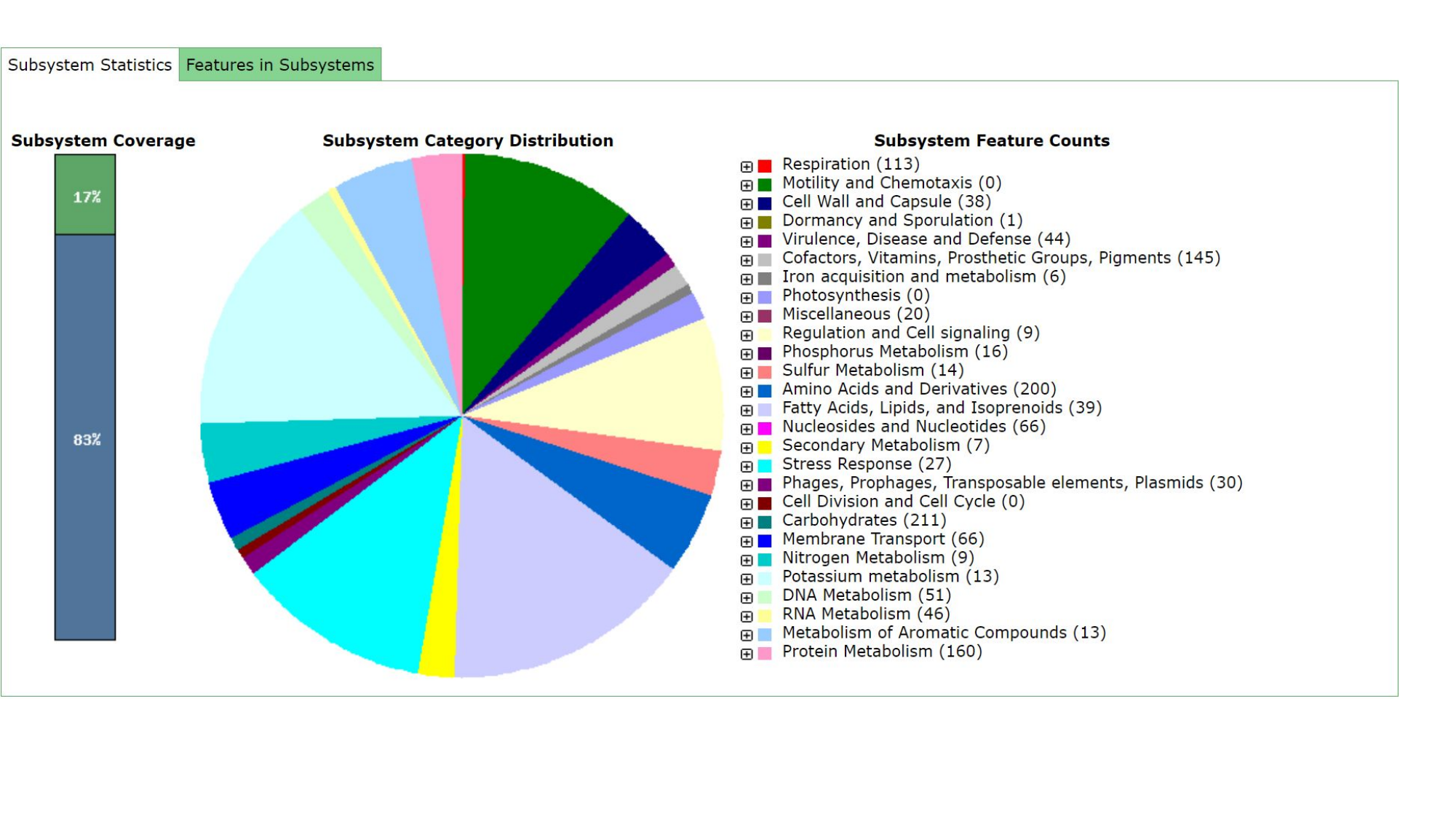

Supplement: Supplementary file 1 [file genes-15-01048-s001.zip › Supp. Figure S3.pptx]

## Slide 1
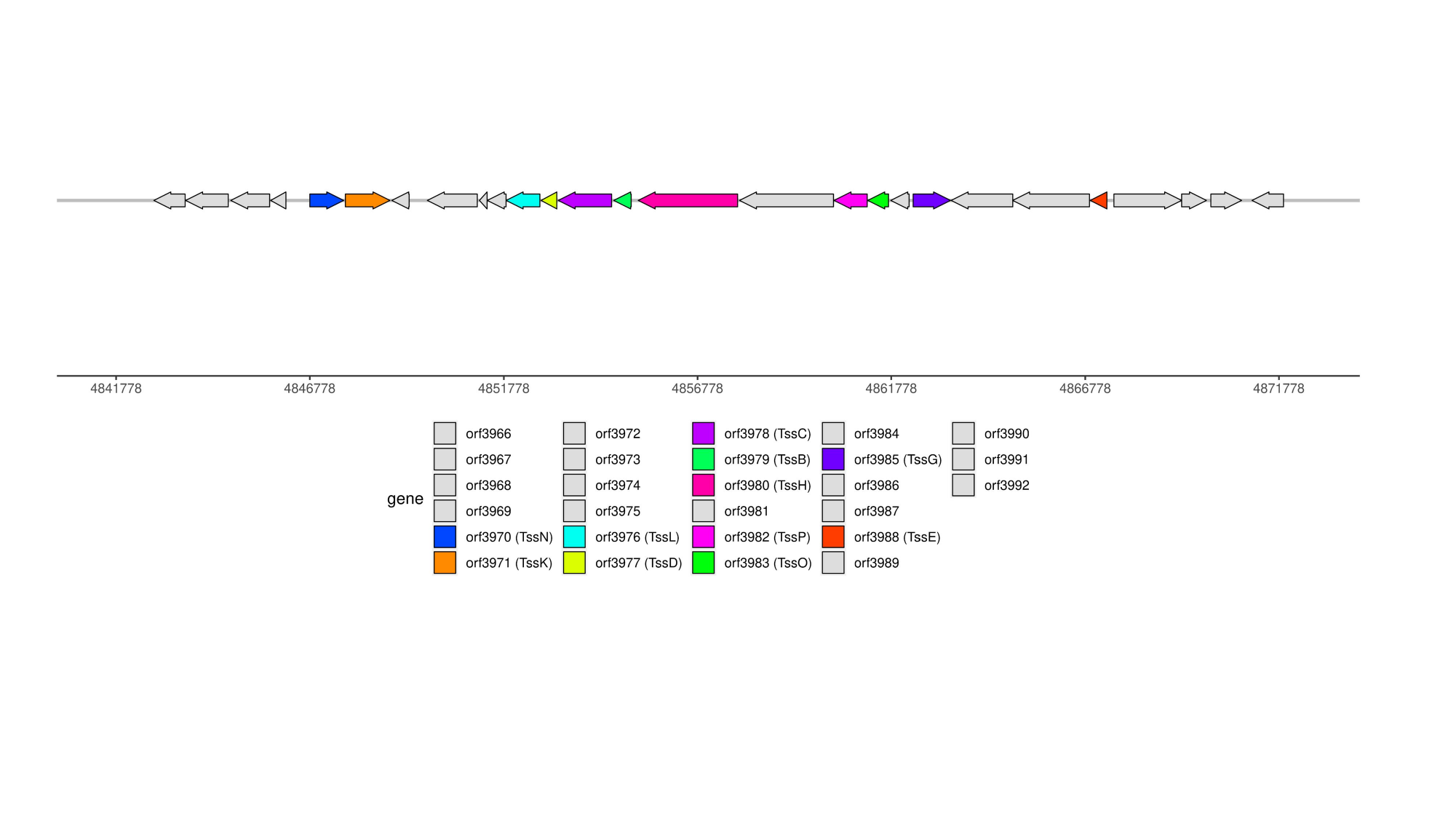

Supplement: Supplementary file 1 [file genes-15-01048-s001.zip › Supp. Figure S4 predicted T6SS cluster.pptx]

## Slide 1
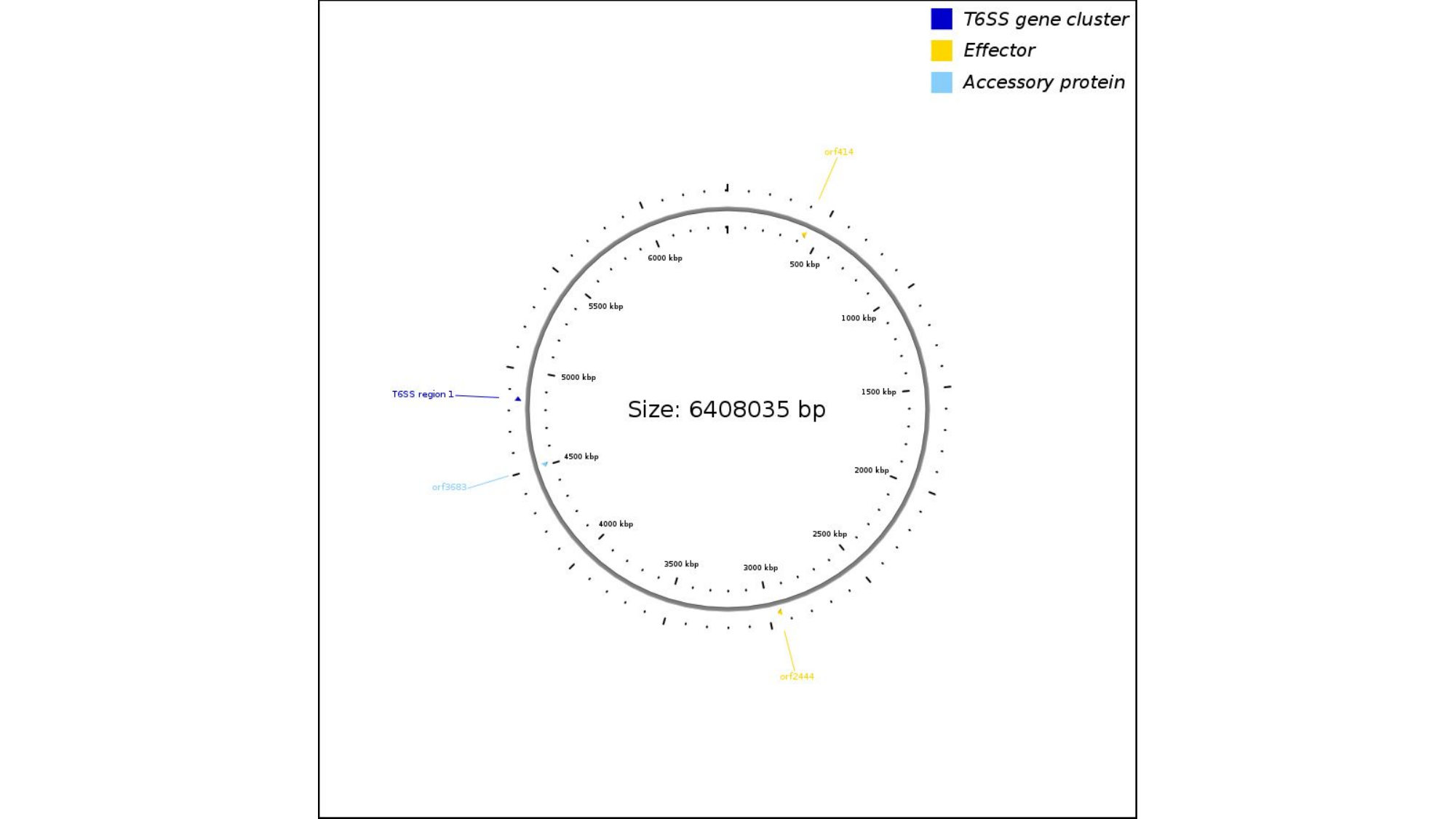

Supplement: Supplementary file 1 [file genes-15-01048-s001.zip › Supp. Figure S5 T6SS location.pptx]

## Slide 1
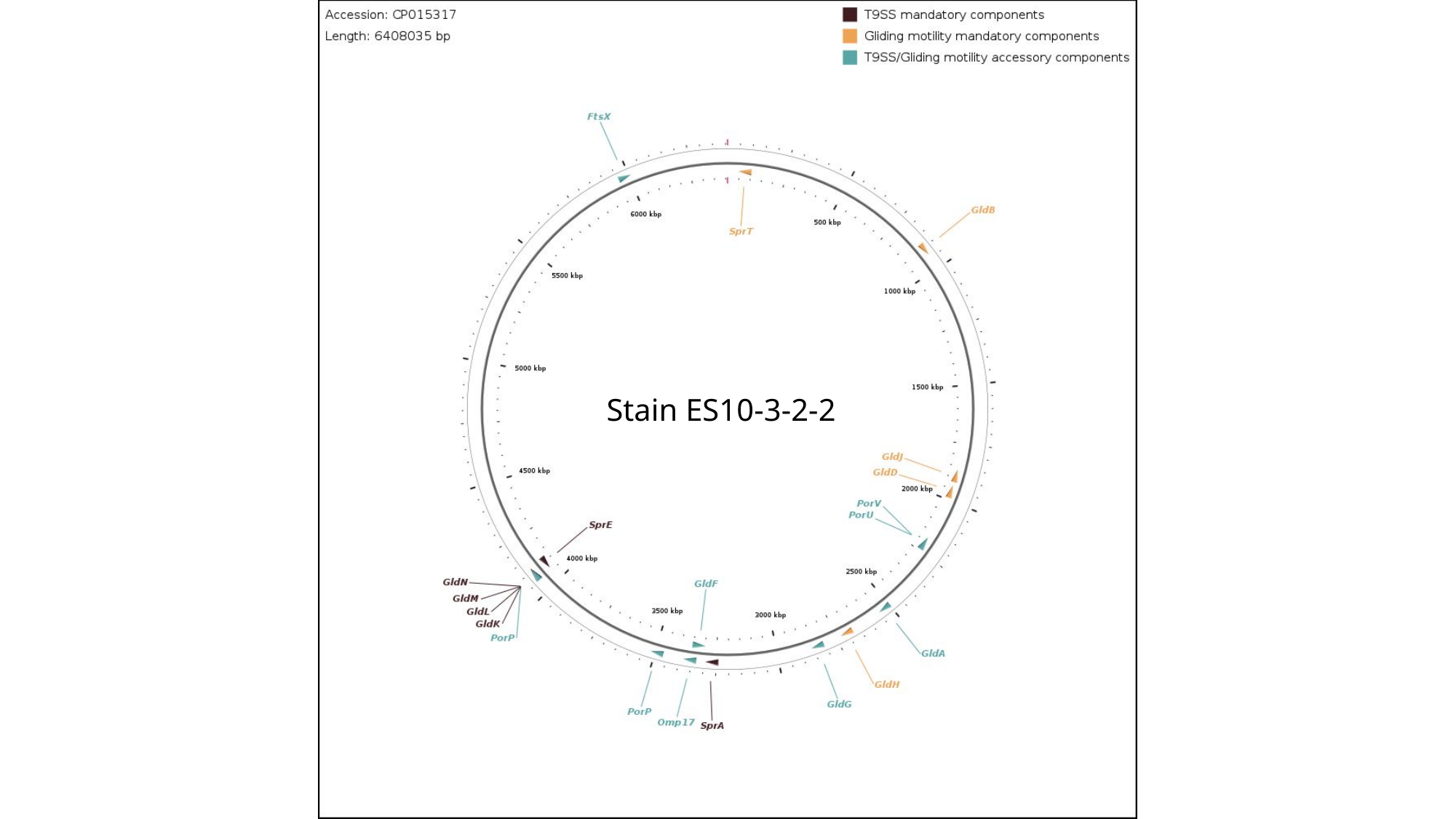

Stain ES10-3-2-2

Supplement: Supplementary file 1 [file genes-15-01048-s001.zip › Supp. Figure S6 T9SS locations.pptx]

## Slide 1
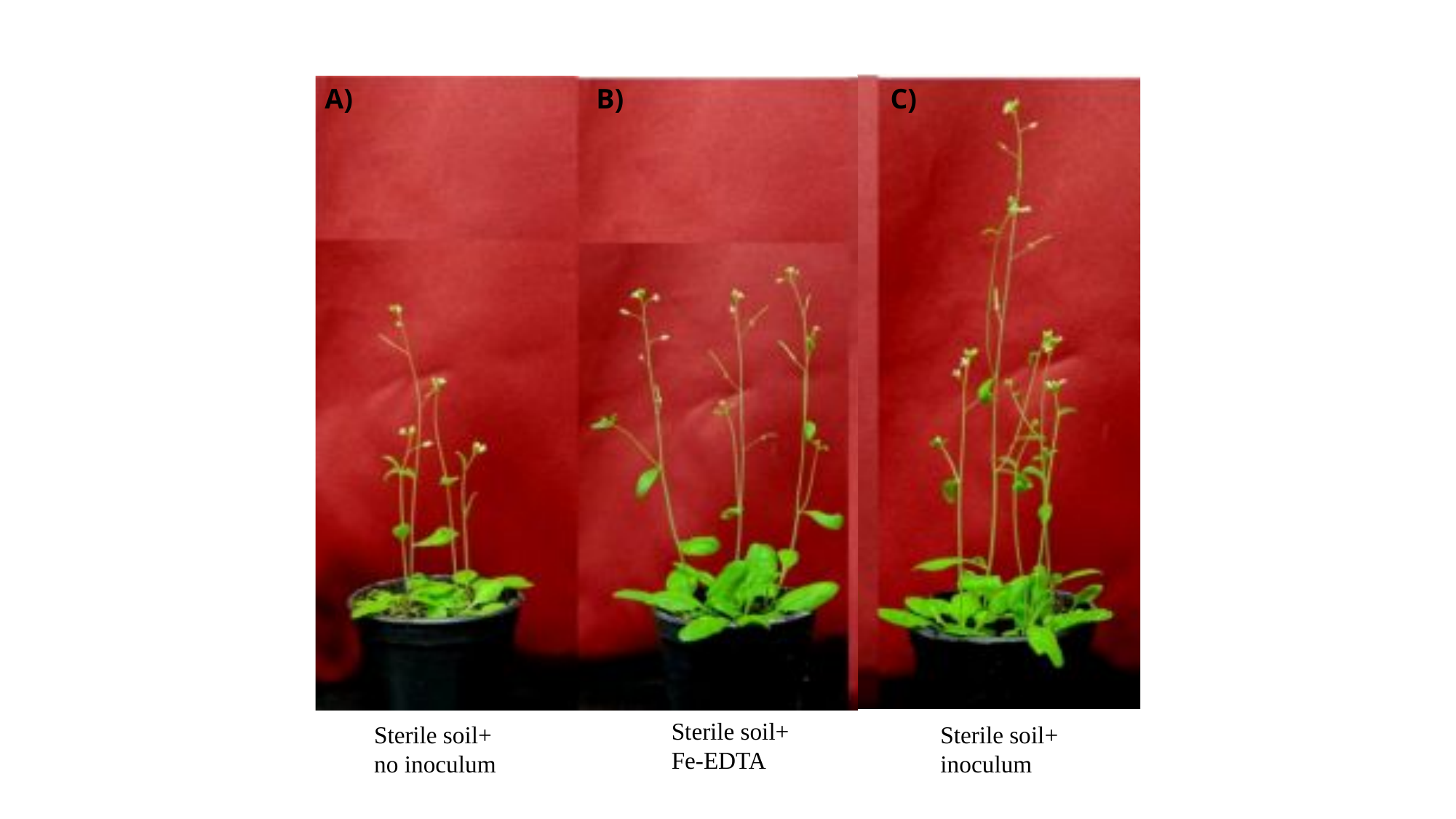

A)
B)
C)
Sterile soil+
Fe-EDTA
Sterile soil+
inoculum
Sterile soil+
no inoculum

Supplement: Supplementary file 1 [file genes-15-01048-s001.zip › Supp. Figure S8 plant.pptx]
